# SS31 Alleviates Pressure Overload-Induced Heart Failure Caused by Sirt3-Mediated Mitochondrial Fusion

**DOI:** 10.3389/fcvm.2022.858594

**Published:** 2022-05-03

**Authors:** Mengying Suo, Yan Qi, Lingxin Liu, Chunmei Zhang, Jingyuan Li, Xuefang Yan, Chen Zhang, Yun Ti, Tongshuai Chen, Peili Bu

**Affiliations:** Key Laboratory of Cardiovascular Remodeling and Function Research, Chinese Ministry of Education, Chinese National Health Commission, Chinese Academy of Medical Sciences, State and Shandong Province Joint Key Laboratory of Translational Cardiovascular Medicine, Department of Cardiology, Qilu Hospital, Cheeloo College of Medicine, Shandong University, Jinan, China

**Keywords:** SS31, heart failure, Sirt3, mitochondrial fusion, myocardial fibroblasts

## Abstract

Heart failure caused by pressure overload is one of the leading causes of heart failure worldwide, but its pathological origin remains poorly understood. It remains critical to discover and find new improvements and treatments for pressure overload-induced heart failure. According to previous studies, mitochondrial dysfunction and myocardial interstitial fibrosis are important mechanisms for the development of heart failure. The oligopeptide Szeto-Schiller Compound 31 (SS31) can specifically interact with the inner mitochondrial membrane and affect the integrity of the inner mitochondrial membrane. Whether SS31 alleviates pressure overload-induced heart failure through the regulation of mitochondrial fusion has not yet been confirmed. We established a pressure-overloaded heart failure mouse model through TAC surgery and found that SS31 can significantly improve cardiac function, reduce myocardial interstitial fibrosis, and increase the expression of optic atrophy-associated protein 1 (OPA1), a key protein in mitochondrial fusion. Interestingly, the role of SS31 in improving heart failure and reducing fibrosis is inseparable from the presence of sirtuin3 (Sirt3). We found that in Sirt3KO mice and fibroblasts, the effects of SS31 on improving heart failure and improving fibroblast transdifferentiation were disappeared. Likewise, Sirt3 has direct interactions with proteins critical for mitochondrial fission and fusion. We found that SS31 failed to increase OPA1 expression in both Sirt3KO mice and fibroblasts. Thus, SS31 can alleviate pressure overload-induced heart failure through Sirt3-mediated mitochondrial fusion. This study provides new directions and drug options for the clinical treatment of heart failure caused by pressure overload.

## Introduction

Millions of patients worldwide suffer from hypertension. Hypertension and its cardiovascular sequelae, left ventricular (LV) hypertrophy and congestive heart failure directly or indirectly accounts for inordinate morbidity and mortality globally (1). Hypertension-induced left ventricular pressure overload-induced left ventricular hypertrophy and failure is one of the important causes of heart failure worldwide, which has put tremendous pressure on human health and social economy. Although several signaling pathways have been known to influence cardiac myocyte hypertrophy, the precise molecular pathogenesis of LV hypertrophy and failure in response to pressure overload remains unclear (2). Studies have shown that myocardial interstitial fibrosis and mitochondrial dysfunction are important mechanisms for the development of HF (3). Although the pathophysiology is complicated, mitochondrial dysfunction seems to be an important therapeutic target for directly improving heart function (4).

In recent years, studies have shown that the process of mitochondrial dynamics, which refers to the coordinated cycle of biogenesis, fusion, fission, and degradation, is becoming a core participant in cardiovascular homeostasis (5). Enhancing mitochondrial fusion and reducing fission can improve heart function (6). As an essential protein for mitochondrial fusion, Optic Atrophy 1 (Opa1) has also received increasing attention for its role in improving cardiovascular diseases. Studies have shown that the down-regulation of Opa1 will accelerate the progression of HF. Based on previous research, we learned that the Szeto-Schiller (SS)-31 peptide (D-Arg-2‘,6‘-dimethyltyrosine-Lys-Phe-NH2) belongs to a family of aromatic cationic peptides, has beneficial effects on myocardial injury, neurodegenerative injury and diabetic nephropathy (7, 8). SS31 selectively target to mitochondrial inner membrane and can scavenge superoxide, hydrogen peroxide, peroxynitrite and hydroxyl radicals (9), so that it has a significant effect on improving Ang-induced cardiac hypertrophy, fibrosis and apoptosis, and saving the HF phenotype of Gαq overexpression mice (7). However, studies have not yet confirmed the effect of SS31 on pressure-overloaded heart failure. Whether SS31 can improve pressure-loaded heart failure, and whether SS31 can improve HF through OPA1 also needs further research.

As we all know, Sirt3 is a highly conserved nicotinamide adenine dinucleotide (NAD) dependent deacetylase, which plays an important role in regulating cellular stress responses, metabolism, growth and apoptosis (10). Sirt3 can affect the physiological processes of mitochondria, including oxidative pressure, calcium overload, mitochondrial apoptosis and production (11). More and more studies have shown that Sirt3 can directly interact with the key proteins of mitochondrial fission and fusion. And through the interaction between these proteins, can provide new targets for the treatment of diseases (12). So, is the role of OPA1 also affected by Sirt3 in pressure overload-induced heart failure? Whether the effect of SS31 on HF achieved by affecting the expression of Sirt3/OPA1? Also need us to further research.

Therefore, we used transverse aortic constriction (TAC) technology to establish pressure-overload heart failure models in wild-type and Sirt3-/- knockout mice and partly given the drug SS31 intervention to explore whether SS31 could alleviates pressure overload-induced heart failure caused by Sirt3-mediated mitochondrial fusion.

## Materials and Methods

### Animal Model

All animal studies were approved by the appropriate ethics committee and performed in accordance with the ethical standards specified in the 1964 Declaration of Helsinki and its later amendments. All experiments were approved by the ethics boards of Qilu Hospital of Shandong University. Mice were purchased from the Jackson Laboratory. We use TAC surgery to construct a mouse HF model. The survival rate after TAC is 50%. Then the surviving mice were divided into six groups (*n* = 5): (1) WT; (2) WT + TAC; (3) WT + TAC + SS31; (4) Sirt3KO; (5) Sirt3KO + TAC; (6) Sirt3KO+ TAC+ SS31. (3) and (6) are administered SS31 (Topscience, Shanghai, China) 3 mg/kg/day for 60 days. Others were given saline as a control. The experimental group was given SS31 by intraperitoneal injection at a dose of 3 mg/kg/d for 60 days, and the sham operation group was given the same volume of normal saline control at the same site.

### Echocardiography

The mice were given isoflurane (1–3%) continuous vaporization inhalation anesthesia, and by controlling the concentration of isoflurane to control the heart rate. Use a mouse-specific ultrasound probe (30 MHz) to perform M-mode, two-dimensional echocardiography and pulse wave doppler (PWD) on mice at least 3 times (Vevo 770, Canada). A four-chamber apical section was used to measure the diastolic function echocardiogram of mice at the level of the mitral valve.

### Histopathology and Immunostaining

The heart tissues were fixed with 4% paraformaldehyde in phosphate-buffered saline at room temperature for 48 h and embedded in paraffin. The heart tissues were cut into 5 μm sections for the following analyses. Masson's trichrome staining was used to measure interstitial and perivascular fibrosis. The cardiomyocyte cell area and fibrotic level were quantified by Image J. The slides were incubated overnight at 4°C with the specific primary antibodies against collagen I(Abcam, ab34710), collagen III (Abcam, ab184993), α-SMA (Abcam, ab7817), TGF-β (Abcam, ab92486) and OPA1 (Abcam, ab157457) (dilution, 1:200) and Sirt3 (Santa Cruz Biotechnology, sc-365175) (dilution, 1:50) for immunohistochemistry.

### Cardiac Fibroblasts Isolation, Culture, Transfection and Treatment

WT and SIRT3-KO fibroblasts were extracted from 1 to 3 days WT and SIRT3-/- mice, respectively. The 1–3 days WT and SIRT3-/- mice were sterilized twice with 75% alcohol and then operated in a sterile environment. The ultra-clean table was opened in the left side of the sternum of the mice. The ventricular tissues of newborn Suckling mice were taken, and the excess blood, clots and other tissues were washed with 4°C HBSS for 3 times in the six-hole plate to remove them. The above process takes place on the ice. After HBSS was absorbed, collagenase ii (0.8 mg/mL) digestive solution equipped with HBSS was added, sealed with sterile tin foil, and digested in sterile cell incubator at 37°C for 3 times, 1 h each time. The digestion could be accelerated by magnetic stirrer and rotor at a speed of 1 r/s. The digestive supernatant was collected three times and centrifuged at 800 r/min for 5 min. The cell components at the bottom of the tube were collected. The fibroblasts were collected by differential adherent method and cultured in Dulbecco's Modified Eagle Medium (DMEM) (13). The cell suspensions were plated in a 5% CO2 humidified incubator at 37°C. Some fibroblasts were treated with Ang II (10–7 mmol/L) for 48 h. The lentivirus of Sirt3 was purchased from Shanghai Genechem Co., LTD. In the SS31 treatment group, after the cell starvation treatment, the corresponding concentration of angiotensin II and SS31 (SS31 (Topscience, Shanghai, China) dissolved medium was added to each well, and stimulated for 48 h.

### Immunofluorescence Staining

Fibroblasts were fixed in 4% paraformaldehyde for 20 min, followed by permeabilization with 0.2% Triton X-100 in PBS for 10 min. After blocking, fibroblasts were incubated overnight with anti- α-SMA (Abcam, ab7817) antibody at 4°C, then incubated with fluorescent secondary antibody (1:200, diluted with PBS) in a humidified box at 37°C for 1 h. DAPI was used to stain nuclei. Fluorescence mounting medium was used to mount coverslips. Images were taken with fluorescence microscope (URFLT, Japan Olympus), and analyzed using Image Pro Plus (14).

### Transmission Electron Microscope (TEM)

Gently separate fresh ventricular tissue (2 × 2 mm). Fix with pre-cooled 2.5% glutaraldehyde phosphate buffer (0.1 M, pH 7.4) at 4°C in the dark for 2 days. After washing 5 times in PBS buffer, the tissue samples were placed in 1% osmium tetroxide, dehydrated to 100% with a series of gradient ethanol, and infiltrated into the embedding medium with propylene oxide. Then high-quality ultra-thin sections and ultra-thin sections were cut out for observation, observed under the JEOL JEM-1230 transmission electron microscope. Use ImageJ to analyze digital images.

### Determination of Mitochondria Using the Mito-Tracker Red CMXRos Probe

Mito-Tracker Red CMXRos can specifically label active mitochondria. To observe the morphological structure of mitochondria of the fibroblasts, we used this reagent to label, and then observed under a fluorescence microscope.

### Western Blot Analysis

Proteins were harvested from freshly dissected mouse hearts using cell lysis buffer, the membranes were incubated overnight with the primary antibodies against OPA1, Sirt3 and tubulin (Abcam, ab6046), and incubated with secondary antibodies (1:5000) for 1 h at room temperature. Protein bands were visualized with enhanced chemiluminescence (Millipore, USA) whereas protein levels were detected using chemiluminescence reader (Amersham Imager 600, USA). Relative protein levels were quantified using Image J software.

### Statistical Analysis

All experimental data were obtained from 3 or more independent repeated experiments and were processed by GraphPad prism 8 software package. All data are represented as mean ± SEM. Comparison of multiple groups by one-way ANOVA with Tukey's *post-hoc* test. All statistical tests were two-tailed, and *p* < 0.05 was considered statistically significant.

## Results

### SS31 Improves the Cardiac Hypertrophy and Function, Which Disappears in Sirt3 Knockout Mice

**Figure 1:**
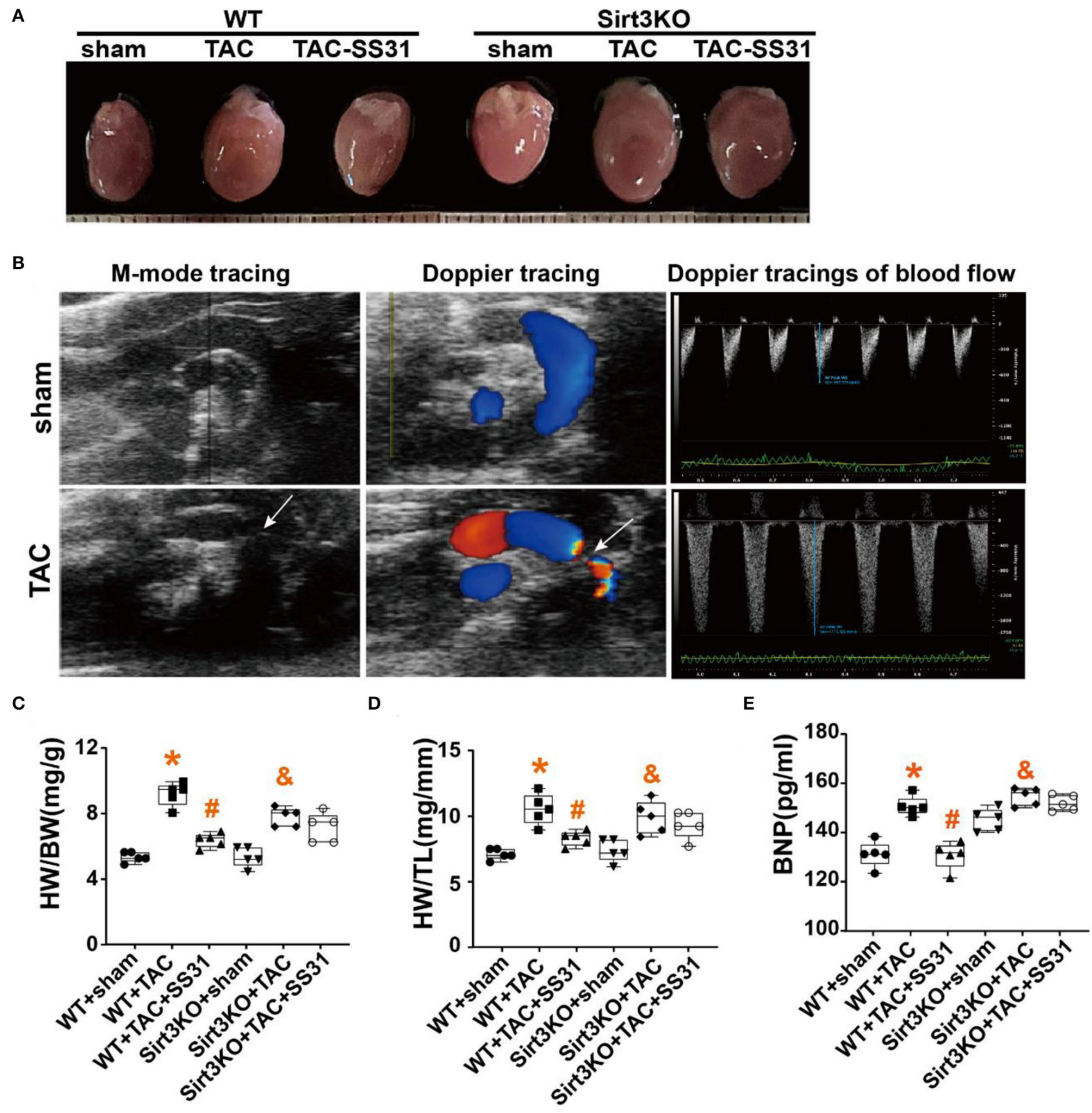
The effect of SS31 on heart function. **(A)** Representative size of the mice heart in different groups; **(B)** Echocardiography was performed on the mice with sham or TAC surgery, Representative M-mode tracing shows the constriction of the aortic arch (white arrow), and the Doppler ultrasound image shows the blood flow and blood flow velocity; **(C)** the ratio of heart weight (mg) to body weight (g) (HW/BW); **(D)** Heart weight to tibia length ratio (HW/TL). **(E)** The level of serum brain natriuretic peptide (BNP) in mice detected by ELISA. (The results are expressed as the means ± SEM, *n* = 5;*p < 0.05 vs. WT + sham group; ^#^*p* < 0.05 vs. WT + TAC group; ^&^*p* < 0.05 vs. Sirt3KO + sham group).

In wild-type and Sirt3KO mice, we performed TAC operation or sham operation to establish pressure overload-induced heart failure model groups (WT-TAC group and Sirt3KO-TAC group) and control groups (WT-sham group and Sirt3-KO-sham group). In the TAC operation group mice, the heart volume (Figure 1A; Table 1) and the heart weight (HW, Table 1) increased; the echocardiography showed that the aortic root (Ao Root) significantly expanded, left ventricular volume (LV Vol) and diastolic septal thickness (IVS; d) increased, and the aortic arch was significantly narrowed in M-mode, but tibia length (TL) did not change significantly (Table 1). The ratio of heart weight to body weight (HW/BW) and heart weight to tibia length ratio (HW/TL) showed a significant increase in the TAC operation group (Figures 1C,D).

**Table 1:** SS31 improves the related indexes of heart structure and function in wild-type mice.

	**WT-sham**	**WT-TAC**	**WT-TAC-SS31**	**Sirt3KO-sham**	**Sirt3KO-TAC**	**Sirt3KO-TAC-SS31**
BW, g	26.7 ± 1.2	21.2 ± 0.79^*^	26.2 ± 0.44^#^	27.0 ± 1.4	23.7 ± 0.85	24.1 ± 0.38
HW, mg	143 ± 3.3	200 ± 8.2^*^	163 ± 4.9^#^	143 ± 6.1	186 ± 7.4^&^	168 ± 8.7
TL, mm	20.0 ± 0.20	19.0 ± 0.32	20.0 ± 0.033	19.5 ± 0.34	19.0 ± 0.29	19.5 ± 0.29
EF%	68.6 ± 2.2	42.3 ± 1.8^*^	59.2 ± 2.7^#^	52.3 ± 2.9	24.34 ± 1.5^&^	35.5 ± 2.4
FS%	39.5 ± 1.5	20.4 ± 1.1^*^	30.6 ± 1.8^#^	26.3 ± 1.8	11.0 ± 0.73^&^	17.4 ± 1.3
E/A	1.69 ± 0.05	1.11 ± 0.04^*^	1.44 ± 0.08^#^	1.44 ± 0.09	0.99 ± 0.04^&^	1.01 ± 0.09
E'/A'	1.43 ± 0.080	0.876 ± 0.055^*^	1.41 ± 0.11^#^	1.60 ± 0.12	0.911 ± 0.10^&^	0.906 ± 0.15
Ao Root, mm	1.15 ± 0.022	1.36 ± 0.038^*^	1.28 ± 0.0092^#^	1.22 ± 0.027	1.38 ± 0.033^&^	1.33 ± 0.019
LV; vol, ul	14.7 ± 1.39	32.4 ± 1.7^*^	16.5 ± 2.7^#^	25.7 ± 3.0	42.5 ± 4.6^&^	31.6 ± 4.5
Lvs, d, mm	0.695 ± 0.045	1.09 ± 0.024^*^	0.684 ± 0.047^#^	0.737 ± 0.050	1.06 ± 0.13^&^	0.772 ± 0.055

*HW, heart weight; BW, body weight; TL, tibia length; EF%, ejection fraction; FS%, fractional shortening; E, maximum ventricular filling velocity in early diastole; A, maximum ventricular filling velocity in late diastole degree; E′, the tissue movement speed of the posterior wall of the left ventricle in the early diastole; A′, the tissue movement speed of the posterior wall of the left ventricle in the late diastole; Ao Root, Aortic root width; LV; vol, left ventricular volume; IVS; d, diastolic interventricular septum thickness. (The results are expressed as the means ± SEM, n = 6*;

* *p < 0.05 vs. WT + sham group*;

# *p < 0.05 vs. WT + TAC group*;

& *p < 0.05 vs. Sirt3KO + sham group)*.

Doppler ultrasound showed that the blood flow at the constricted part of the aortic root of the mice in the TAC operation group obviously disordered, and the flow velocity significantly increased (Figure 1B), while the left ventricular ejection fraction (EF%), left ventricular short axis shortening rate (FS%), E/A and E'/A' significantly reduced (Table 1). Serum of the mice was detected by ELISA, the results showed that the BNP level in the TAC operation group significantly increased (Figure 1E).

In Sirt3KO mice, compared with the TAC operation group, after SS31 intervention, the weight of the mouse heart was not significantly reduced, the heart volume (Figure 1A), the degree of aortic root expansion, the volume of the left ventricle, and the ventricular septal thickness did not decrease (Table 1), and the ratio of HW/BW and HW/TL did not differ significantly (Figures 1C,D). Similarly, in Sirt3KO mice, compared with the TAC operation group, SS31 intervention did not significantly improve EF%, FS%, E/A, E/e' (Table 1), or reduce the BNP level (Figure 1E).

### SS31 Reduces the Severity of Cardiac Fibrosis, Which Disappears in Sirt3 Knockout Mice

**Figure 2:**
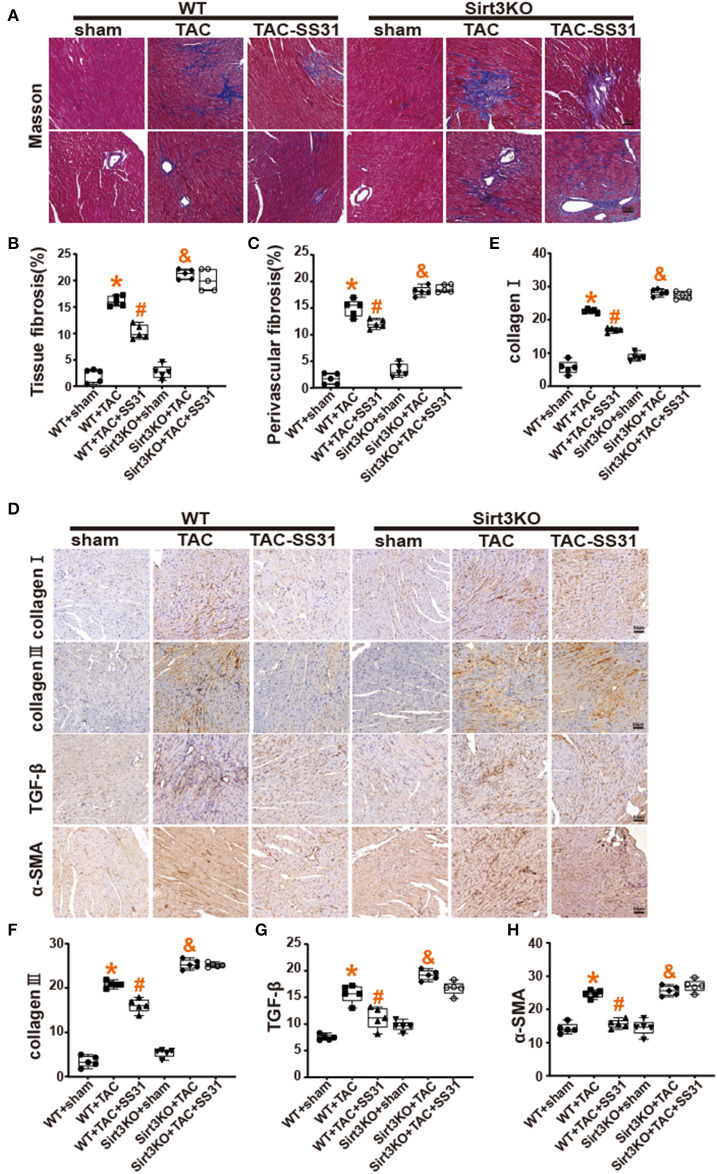
The effect of SS31 on myocardial fibrosis in mice. **(A)** Masson staining images of each group; **(B)** Masson staining statistical analysis of the degree of fibrosis between tissues; **(C)** Masson staining statistical analysis of the degree of perivascular fibrosis (*n* = 5, *p < 0.05, WT + TAC vs. WT + sham; ^#^*p* < 0.05, WT + TAC + SS31 vs. WT + TAC; ^&^*p* < 0.05, Sirt3KO + TAC vs. Sirt3KO + sham); **(D)** Immunohistochemical staining images of collagen I, collagen III, TGF-β and α-SMA in each group (bar = 20 μm); **(E–H)** collagen I and collagen III immunohistochemical staining statistical analysis. (The results are expressed as the means ± SEM, *n* = 5. The one-way ANOVA test was used, and n represents the number of independent experiments. *p < 0.05 vs. WT + sham group; ^#^*p* < 0.05 vs. WT + TAC group; ^&^*p* < 0.05 vs. Sirt3KO + sham group).

We performed modified Masson staining (Figure 2A) and immunohistochemical staining (Figure 2D). The results showed that the collagen fibers around the heart tissue and blood vessels of the mice after TAC were significantly increased (Figures 2B,C), the fiber arrangement disordered, and the density of collagen I and collagen III expression positive areas significantly increased (Figures 2E,F). At the same time, we found that in WT mice, in the SS31 treatment group, the range of myocardial fibrosis in mice significantly reduced, the degree of disorder in the fibrotic area was also significantly alleviated, and the density of collagen I and collagen III expression positive areas significantly reduced. However, in Sirt3KO mice, no improvement in the degree of cardiac fibrosis was observed in the SS31 administration group.

Immunohistochemical staining of TGF-β and α-SMA, the specific markers of myofibroblasts (Figures 2G,H), found that the density of TGF-β and α-SMA expression in the heart tissue of mice in the TAC operation group was significantly increased. In WT mice, the density of TGF-β and α-SMA positive areas decreased in the SS31 treatment group, but in Sirt3KO mice, no significant changes were observed in the SS31 treatment group. The intervention of SS31 can significantly inhibit transdifferentiation, reduce the degree of myocardial fibrosis, and play a role in protecting the heart in the process of pressure overload-induced heart failure. However, the role of SS31 depends on the existence of Sirt3.

### SS31 Improves the Morphological Structure of Myocardial Mitochondria and Increases the Expression of OPA1 Protein, Which Disappears in Sirt3 Knockout Mice

**Figure 3:**
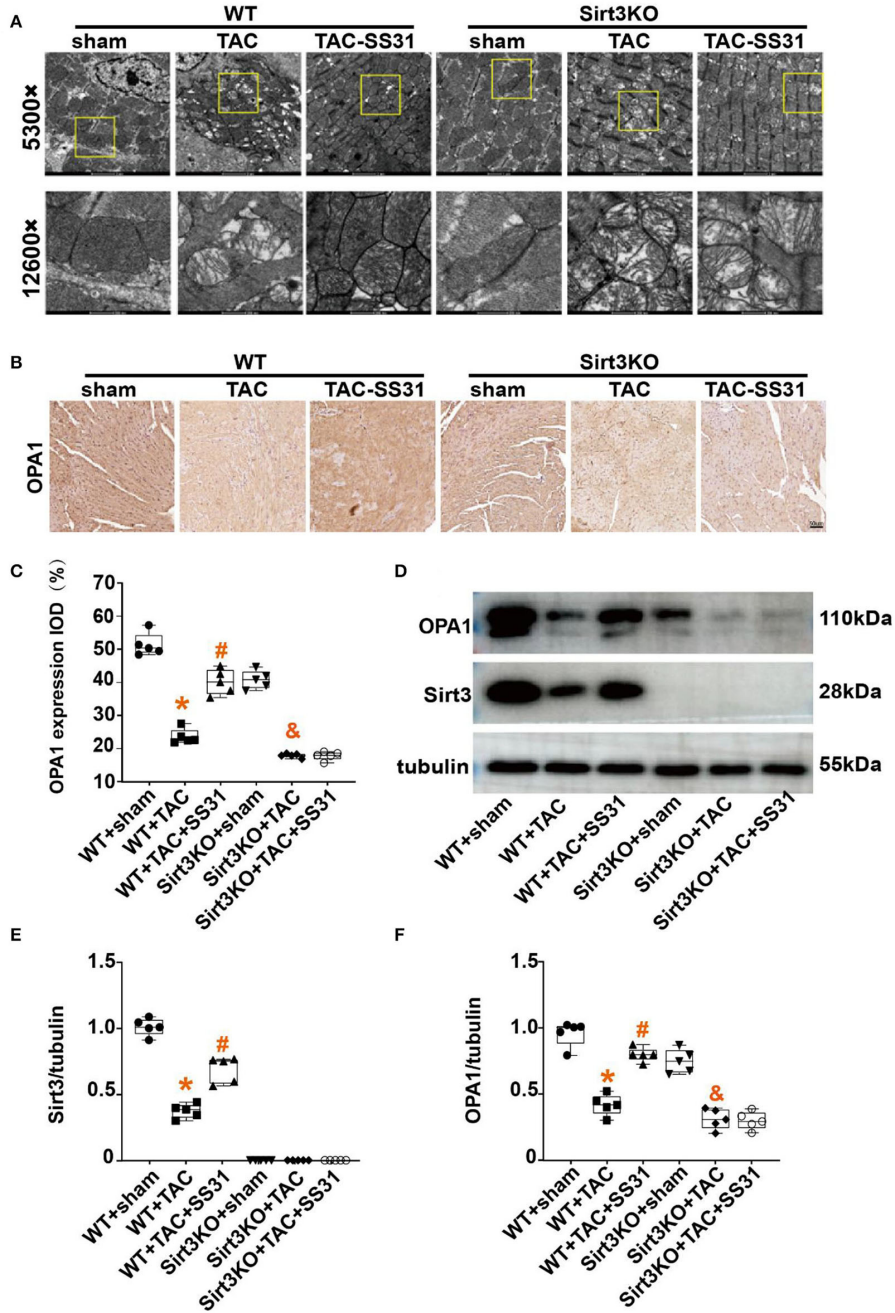
The effect of SS31 on the morphological structure of mouse heart mitochondria. **(A)** Transmission electron microscope (TEM) observation of myocardial tissue in each group at 8 weeks postoperatively. The image above is a representative evaluation of the left ventricle's TEM image. **(B–F)** Observing the effect of SS31 on the expression of mitochondrial fusion protein OPA1 in mouse heart at the molecular level; **(B)** Immunohistochemical staining of OPA1 in mouse myocardial tissue; **(C)** Statistical analysis of OPA1 immunohistochemistry; **(D)** Western blot pictures of Sirt3 and OPA1 of myocardial tissue in mice; **(E)** Western blot statistical analysis of Sirt3; **(F)** Western blot statistical analysis of OPA1. (The results are expressed as the means ± SEM, *n* = 5. The one-way ANOVA test was used, and n represents the number of independent experiments. *p < 0.05 vs. WT + sham group; ^#^*p* < 0.05 vs. WT + TAC group; ^&^*p* < 0.05 vs. Sirt3KO + sham group).

We conducted transmission electron microscopic observations on the mitochondrial structure in the heart tissue (Figure 3A), and performed statistics on the Mean Branch Length, the Mean Area, the Mean Form Factor and the Mean Perimeter of mitochondria (Supplementary Figures 1E–H). The results showed that after TAC, the mitochondria of myocardial cells of the mice were significantly broken, arranged disorderly, and apoptotic vacuoles increased, especially the mitochondrial cristae were significantly reduced, the cristae arrangement was disordered, and the gaps increased. After treatment with SS31, wild-type mice can partially restore the ultrastructure, volume and number of mitochondria, and the structure of mitochondrial cristae is also partially restored. However, in Sirt3KO mice, the ultrastructure of myocardial mitochondria did not improve.

Mitochondrial fusion is an important mechanism affecting HF, we used immunohistochemistry (Figure 3B) and Western Blot (Figure 3D) to detect the expression of OPA1. The results showed (Figures 3C,F) that compared with the sham operation group, the OPA1 protein expression level in the heart tissue of the TAC operation group was significantly reduced. In wild-type mice, SS31 treatment increased the expression of OPA1, indicating that SS31 improved mitochondrial fusion by affecting OPA1. In Sirt3KO mice, SS31 treatment did not significantly change the expression of OPA1. It shows that the influence of SS31 on OPA1 is mediated by Sirt3. The results of Western Blot (Figures 3D,E) also showed that TAC surgery significantly reduced the expression of Sirt3 in the mouse heart, and SS31 treatment could significantly promote the expression of Sirt3.

### SS31 Inhibits the Transdifferentiation of Fibroblasts Induced by Ang II, Which Disappears in Sirt3 Knockout Fibroblasts

**Figure 4:**
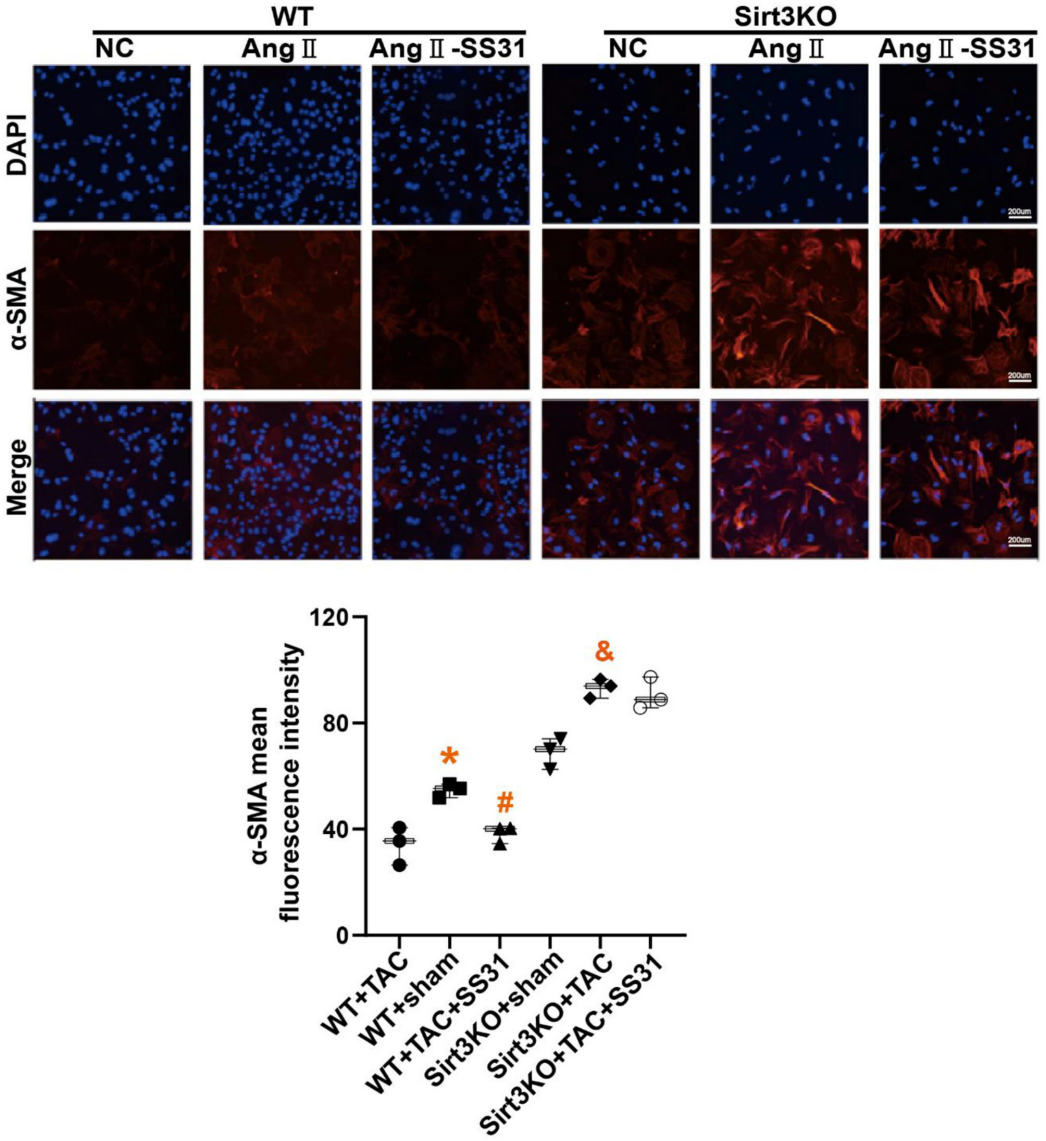
SS31 inhibits the transdifferentiation of cardiac fibroblasts induced by angiotensin II. The expression of α-SMA in fibroblasts was shown by immunofluorescence staining (red). DAPI stains the nucleus (blue). *p < 0.05. WT+TAC vs. WT+sham, *#p* < 0.05. WT+TAC+SS31 vs. WT+TAC, &*p* < 0.05. Sirt3KO+TAC vs. Sirt3KO+sham.

Cardiac fibroblasts (CFs) from WT and Sirt3KO suckling mice were extracted and stimulated with Ang II to induce the transdifferentiation of fibroblasts into myofibroblasts. We used immunofluorescence to identify the expression level of α-SMA (Figure 4), a marker for the transdifferentiation of fibroblasts into myofibroblasts. We found that in myocardial fibroblasts extracted from WT neonatal mice, fibroblasts transdifferentiated into myofibroblasts after stimulation by Ang II, while the treatment of SS31 attenuated fibroblast transdifferentiation induced by Ang II. However, SS31 treatment could not attenuate the transdifferentiation of Sirt3KO cardiac fibroblasts induced by Ang II.

### SS31 Promotes Mitochondrial Fusion and OPA1 Protein Expression in Fibroblasts Induced by Ang II, Which Disappears in Sirt3 Knockout Fibroblasts

**Figure 5:**
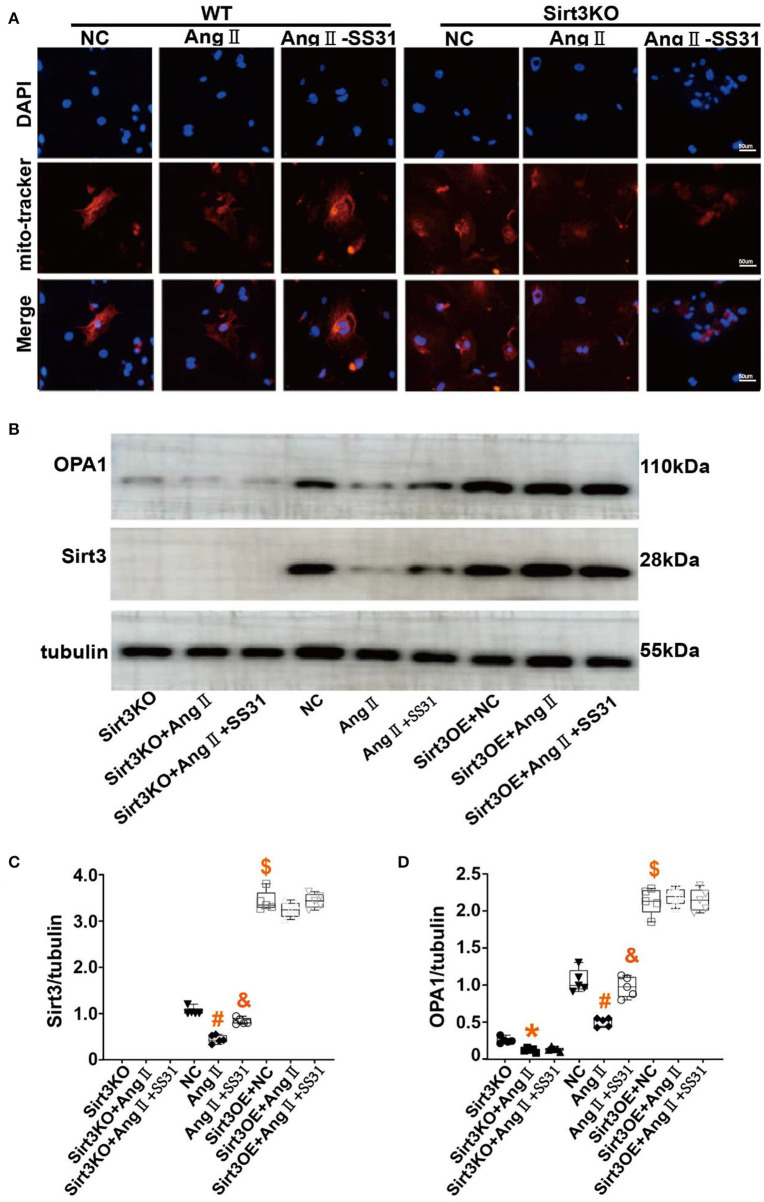
SS31 regulates mitochondrial fusion in fibroblasts. **(A)** Immunofluorescence staining of mitochondria in cells to observe the morphology of mitochondria. Red represents mitochondria and blue represents the nucleus. **(B–D)** Western Blot to observe the role of SS31 in the transdifferentiation of cardiac fibroblasts. **(B)** Western blot pictures of Sirt3 and OPA1 between different groups; **(C)** Western blot statistical analysis of Sirt3; **(D)** Western blot statistical analysis of OPA1. (The results are expressed as the means ± SEM, *n* = 3. The one-way ANOVA test was used, and n represents the number of independent experiments. *p < 0.05 vs. Sirt3KO group; ^#^*p* < 0.05 vs. WT + NC group; ^&^*p* < 0.05 vs. WT + Ang II group; ^$^*p* < 0.05, Sirt3OE vs. WT + NC group).

In WT mouse cardiac fibroblasts, the mitochondrial network is mostly filamentous and branched, and there are few fragmented or spherical mitochondria. On the contrary, Ang II-induced cardiac fibroblasts have more fragments and globular mitochondria. After SS31 treatment, fragmented or spherical mitochondria decreased, and the proportion of filamentous and branched mitochondria increased. In Sirt3KO cardiac fibroblasts, the therapeutic effect of SS31 disappeared (Figure 5A). To determine whether the protective effect of SS31 on cardiomyocyte fibroblast transdifferentiation is related to mitochondrial fusion and whether it is Sirt3-dependent, we measured the expression of OPA1 and Sirt3 in WT fibroblasts and Sirt3KO fibroblasts induced by Ang II. Compared with the control group, the expression of Sirt3 and OPA1 decreased after Ang II induction, suggesting a decrease in mitochondrial fusion. In fibroblasts extracted from WT mice, after SS31 treatment, the levels of Sirt3 and OPA1 increased significantly, but there was no such change in Sirt3KO fibroblasts. When Sirt3 is overexpressed in fibroblasts, the inhibitory effect of Ang II on mitochondrial fusion disappears, which also proves that Sirt3 has the effect of protecting mitochondrial fusion (Figures 5B–D).

## Discussion

Pressure-loaded heart failure is one of the most common types of HF, and it is an important reason for the increased risk of death in the elderly. Many cardiovascular diseases will eventually lead to pressure-load heart failure, such as hypertension (15), severe aortic stenosis (16) and so on. However, we cannot eradicate the occurrence of HF at present. What we can and must do is to delay its progress. Existing studies show that the pathogenesis of pressure-loaded heart failure is very complicated and there is a lack of targeted intervention measures. There was an article reported the global attenuation of TAC-induced proteomic alterations by the mitochondrial targeted peptide SS-31 suggests that perturbed mitochondrial function may be an upstream signal to many of pathway alterations in TAC and supports the potential clinical application of mitochondrial-targeted peptide drugs for the treatment heart failure (17). Myocardial lesions must be closely related to mitochondrial dysfunction, so our research has further explored the effects of pressure-loaded heart failure on mitochondrial dynamics,and explored the therapeutic effect of SS31, which plays an important role in mitochondria.

In fact, mitochondria are essential for the growth and development of eukaryotes. They are cell energy sources (18). Although, classic mitochondrial diseases are rare, but diseases related to mitochondrial dysfunction are very common, especially those caused by environmental factors. For example, mitochondrial dysfunction has been documented in neurodegenerative diseases (19), persistent systemic inflammation (20), cardiovascular disease (21), cancer (22) and diabetes (23). Current studies have shown that pressure loading on the heart leads to myocardial cell damage, lipid peroxidation, overproduction of intracellular reactive oxygen species (ROS) and hydrogen peroxide (22), and mitochondrial ATP production will also be reduced, which will further lead to the occurrence of HF. Then, excessive ROS will cause mitochondrial dysfunction by interacting with mitochondria and cellular components (such as DNA, proteins, and lipids), including abnormalities in mitochondrial dynamics.

Mitochondrial dynamics, including mitochondrial biogenesis, division and fusion (5), play a vital role in ensuring the morphology and integrity of mitochondria. The delicate physiological balance between mitochondrial fission and fusion is essential for maintaining cell growth, regulating cell death, and removing damaged mitochondria (23). After mitochondrial fusion, the shape of the mitochondria is extended and the function of the mitochondria is restored, thereby protecting the cell (24). The key protein OPA1 (25), is located in the inner mitochondrial membrane (IMM) and regulates fusion of the IMM. It also participates in the remodeling of mitochondrial cristae in response to energy pressure or mitochondrial damage, thereby maintaining the integrity and function of the IMM (5). In the occurrence and development of HF, compared with outer membrane fusion, endometrial fusion seems to play a more important role. Therefore, in our research, we mainly studied the effect of SS31 on OPA1.

Sirt3 has an important impact on mitochondrial fission and fusion in cardiovascular disease. In HF, existing research on Sirt3 has mainly focused on cardiomyocytes, and its role in fibroblasts is poorly understood (26). One study shown (27, 28) that Sirt3 could directly deacetylate OPA1, thereby ameliorating myocardial injury. In our study, it was found that the effect of Sirt3 on OPA1 in fibroblasts was not limited to the regulation of acetylation levels, and overexpression of Sirt3 could also directly promote the expression of OPA1 (Figures 5B,D). At the same time, the role of SS31 in HF is inseparable from Sirt3/OPA1. This discovery provides a new understanding of the interaction of Sirt3 and OPA1, and provides a new intervention and therapeutic basis for the treatment of HF.

SS31, as an emerging new therapeutic drug that targets mitochondria and restores the biological functions of mitochondria, is undergoing clinical trials for the treatment of a variety of mitochondrial-related diseases, including aging (29), mitochondrial genetic diseases, ischemia, acute Kidney damage (30) and HF (31). Studies have shown that SS31-mitochondria (Mito) therapy also protects cardiac cells from myocardial ischemia-reperfusion (IR) injury, improves LVEF in IR rats, and inhibits menaquinone-induced markers of oxidative stress (NOX-1, NOX-2, oxidized protein), plays a protective role in ischemic heart failure (32). SS31 is expected to become a new choice for clinical treatment. SS31 can also interact with cardiolipin (CL) in the IMM, and stabilize the interaction between CL, electron transport chain, and complex II, thereby preventing oxidative pressure and release of cytochrome c. CL also interacts with OPA1, which is essential for mitochondrial fusion (33). Our study added that SS31 can significantly increase the expression of Sirt3, and promote the expression of the OPA1 through Sirt3, thereby exerting a protective effect on HF induced by pressure overload. This research also provides a new mechanism for SS31 to be used in the clinical treatment of HF, which is of great significance.

## Data Availability Statement

The original contributions presented in the study are included in the article, further inquiries can be directed to the corresponding author.

## Ethics Statement

The animal study was reviewed and approved by the Ethics Boards of Qilu Hospital of Shandong University.

## Author Contributions

MS and YQ jointly completed this project. TC and PB conceived this work. LL, ChuZ, JL, XY, CheZ, and YT provided checks and proofreading for the project. All authors read and approved the final version of the manuscript.

## Funding

This work was supported by National Key R&D Plan of China [No. 2017YFC1700502], National Natural Science Foundation for Young Scientists of China (Nos. 82100449, 82100279, and 81700366), ECCM Program of Clinical Research Center of Shandong University (No. 2021SDUCRCA004), Natural Science Foundation of Shandong Province (Nos. ZR2019QH010 and ZR2021MH011], and Cardiovascular Multidisciplinary Integrated Research Fund (No. z-2016-23-2101-10).

## Conflict of Interest

The authors declare that the research was conducted in the absence of any commercial or financial relationships that could be construed as a potential conflict of interest.

## Publisher's Note

All claims expressed in this article are solely those of the authors and do not necessarily represent those of their affiliated organizations, or those of the publisher, the editors and the reviewers. Any product that may be evaluated in this article, or claim that may be made by its manufacturer, is not guaranteed or endorsed by the publisher.
