# Supplementary figures and images for: SS31 Alleviates Pressure Overload-Induced Heart Failure Caused by Sirt3-Mediated Mitochondrial Fusion
Source: Front Cardiovasc Med. 2022 May 3;9:858594. doi: 10.3389/fcvm.2022.858594 (PMC9110818; doi:10.3389/fcvm.2022.858594)

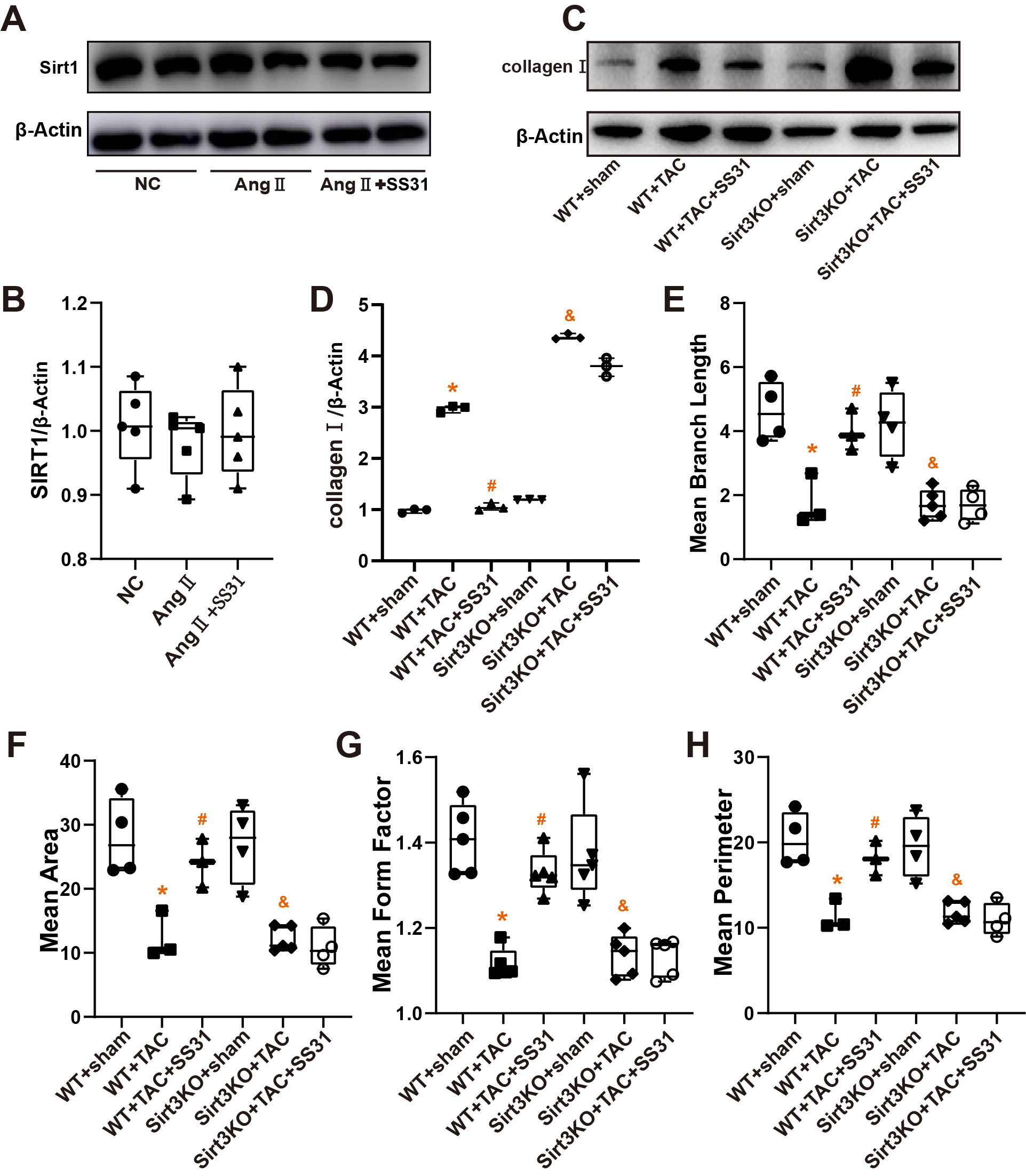

Supplement: Supplementary file 1 [file Figure_1.TIF]
